# A Comparative and Collaborative Study of the Hydrodynamics of Two Swimming Modes Applicable to Dolphins

**DOI:** 10.3390/biomimetics8030311

**Published:** 2023-07-14

**Authors:** Dan Xia, Zhihan Li, Ming Lei, Han Yan, Zilong Zhou

**Affiliations:** School of Mechanical Engineering, Southeast University, Nanjing 211189, China; lzh809963603@163.com (Z.L.); mingleiseu@163.com (M.L.); hanyanseu@gmail.com (H.Y.); 220210297@seu.edu.cn (Z.Z.)

**Keywords:** hydrodynamics, comparison and collaboration, BCF mode, MPF mode, swimming dolphins

## Abstract

This paper presents a hydrodynamics study that examines the comparison and collaboration of two swimming modes relevant to the universality of dolphins. This study utilizes a three-dimensional virtual swimmer model resembling a dolphin, which comprises a body and/or caudal fin (BCF) module, as well as a medium and/or paired fin (MPF) module, each equipped with predetermined kinematics. The manipulation of the dolphin to simulate various swimming modes is achieved through the application of overlapping grids in conjunction with the parallel hole cutting technique. The findings demonstrate that the swimming velocity and thrust attained through the single BCF mode consistently surpass those achieved through the single MPF mode and collaborative mode. Interestingly, the involvement of the MPF mode does not necessarily contribute to performance enhancement. Nevertheless, it is encouraging to note that adjusting the phase difference between the two modes can partially mitigate the limitations associated with the MPF mode. To further investigate the potential advantages of dual-mode collaboration, we conducted experiments by increasing the MPF frequency while keeping the BCF frequency constant, thus introducing the concept of frequency ratio (*β*). In comparison to the single BCF mode, the collaborative mode with a high *β* exhibits superior swimming velocity and thrust. Although its efficiency experiences a slight decrease, it tends to stabilize. The corresponding flow structure indirectly verifies the favorable impact of collaboration.

## 1. Introduction

The complex marine environment contains ever-changing hydrodynamics phenomena that are waiting for humans to challenge. Whales and dolphins, which depend on the marine environment for survival, have many little-known characteristics and exhibit excellent swimming performance. Inspired by dolphins, biomimetic underwater vehicles (BUVs) have been rapidly developed with the promise of explaining hydrodynamic mechanisms and exploiting ocean resources [1,2,3,4]. Unlike submarines that rely on propellers for propulsion [5,6], BUVs can imitate the swimming modes of dolphins, and actively respond to the attached fluid by multifin collaborative movements, thus achieving stable and efficient propulsion. As a leader in ocean sports, dolphins have always been the embodiment of high agility and explosiveness. In a sense, imitating the kinematics of dolphins is considered the best shortcut that can be applied to the design of high-performance BUVs. The inspiration for the current study came from the interaction between the dolphin’s body and various fins, such as the pectoral, dorsal and caudal fins.

Fish swimming modes can be divided into the body and/or caudal fin (BCF) mode and the median and/or paired fin (MPF) mode [7]. Of these, the BCF mode has always been treated as the main enabler for the propulsion system, which has been widely reported in physical experiments [8,9,10,11] and numerical simulations [12,13,14,15]. For example, Feilich and Lauder used a mechanically driven flapping foil to study how different shapes, tail shaft depths and other factors affect swimming performance [11]. While meeting the fixed swing frequency, Park et al. determined the conditions to maximize the thrust generated by the compliant caudal fin propulsion system [9]. Yu et al. applied the multijoint caudal structure and the high-thrust control strategy to a robotic dolphin to achieve high-speed advancement and even high-power leaping movement [16]. Liu used a three-dimensional (3D) low Reynolds number model to analyze tadpoles’ propulsion and revealed that the shape and kinematics of the tadpole work together to create a small “dead water” region between the head and tail of the growing hind limbs [17]. Xia et al. relied solely on the traveling wave propulsion of the body and caudal fin to realize a self-propelled simulation, and obtained a new energy-saving exercise mode [18]. Chung et al. proposed a novel fluid–structure interaction model to simulate the swinging performance of the caudal fin [13]. Regarding the MPF mode, whether in a physical experiment or a numerical simulation, a pair of pectoral fins are installed symmetrically on both sides of the fish body [19,20,21,22,23,24]. In addition, Behbahani and Tan proposed a flexible passive joint to connect the pectoral fins and the fish body, and maximized its effect on propulsive performance by relying on a rapid-bending-recovery mechanism [25]. Xu and Wan used ultra-high-resolution grids to separate the fish and fluid domains, and realized self-propulsion based on the compound flapping of pectoral fins [26]. Bianchi presented a numerical model of the bullnose ray swinging motion and found that the MPF mode enables highly efficient self-propulsion [27]. Li and Ma once proposed a submarine propulsion with flapping wings on both sides, using the collaboration of various wings to improve the propulsion performance, and thus this design had been considered as an innovative application of the MPF mode on submarines [28]. Taken together, it can be found that these two swimming modes, namely, BCF and MPF, have been systematically studied as a single heuristic model; however, the potential superiority arising from their collaboration has been rarely reported.

In previous studies, individual fins were distinguished and studied to help eliminate confusion and clarify their respective quantitative contribution [29,30,31,32], but it is generally believed that the interaction between various parts of the fish body may potentially improve thrust generation and propulsive efficiency [33]. Wang summarized the characteristics of collaborative work of pectoral and caudal fins, and pointed out that there were few studies on multifin collaboration in fish [34]. Tytell used particle image velocimetry to visualize the 3D wake interaction between various fins around the bluegill sunfish and quantified the contribution of each fin to the wake [35]. Li adopted the pufferfish’s multifin deformation model to evaluate the hydrodynamics of its fin–fin system and found that the collaboration of multifins can promote thrust function [36]. Berlinger proposed a biomimetic multifin device and tested the behavioral traits associated with fish [37]. Castano designed a robotic fish driven by rowing pectoral fins, which is executed by a dual-loop controller that simulates the state of multifin movement [38]. These multifin collaborative studies were mainly focused on experiments with finite motion parameters, thus lacking systematic findings. As the main contributors to propulsion, collaborative studies of BCF and MPF modes, especially numerical simulations, have not formed a complete theoretical system yet.

The objective of this work is to compare the contributions of the BCF and MPF modes to propulsion and to further explore the potential superiority arising from collaboration between these two modes. In the traditional BCF or MPF mode, scholars only adopted a single fin function without considering the superposition effect between multiple fins, so the overall performance of the swimmer was not fully utilized. The collaborative mode configured in this work will maximize the integrity and coordination of the swimmer’s behavior. Specifically, a virtual dolphin equipped with a BCF module and a MPF module is selected as the swimmer, and the kinematics of these two modules are configured on the swimmer, respectively. A detailed quantitative method is proposed and implemented to control the collaborative motion between the BCF and MPF modes. The hydrodynamics property and the collaboration superiority of the swimmer are obtained through numerical simulation.

## 2. Materials and Methods

### 2.1. Physical Model

**Figure 1:**
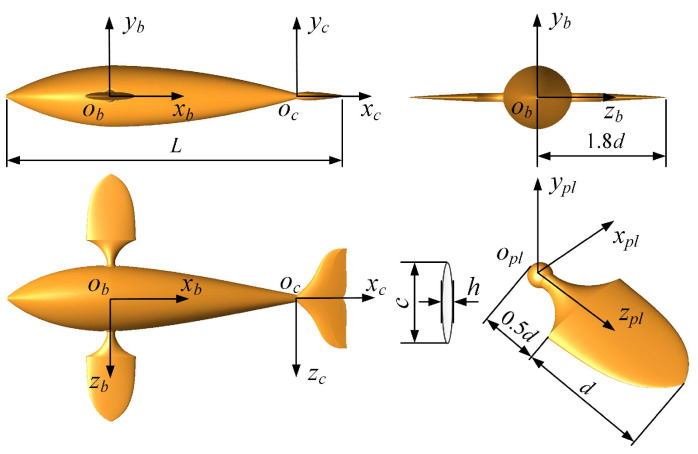
Physical model of the virtual swimmer.

Here, we employed a dolphinlike model as the virtual swimmer. As shown in Figure 1, the swimmer was located in the coordinate system *o_b_x_b_y_b_z_b_*, and it consisted of a smooth main body, a pair of symmetrical pectoral fins and an umbrella-shaped caudal fin. The size of the swimmer was based on a model of a robotic dolphin being developed in our laboratory with a length of *L* = 0.3 m. Regarding the design of the pectoral fins on both sides, we installed a handle at the root of each pectoral fin and embedded it into the side of the body. Taking the left pectoral fin as an example, we defined the coordinate system *o_pl_x_pl_y_pl_z_pl_* to describe its position. The left pectoral fin in the *o_pl_x_pl_y_pl_* plane was simplified to an elliptical cross-section with width *c* = 0.15*L*, thickness *h* = 0.025*L* and chord length *d* = 0.2*L*, and the length of the handle was 0.5*d*. The length from the center of mass of the swimmer to the tips of the pectoral fins was 1.8*d*. The cross-section of the caudal fin was an NACA0024-shaped airfoil, and its active deformation was updated in the coordinate system *o_c_x_c_y_c_z_c_*. It is necessary to note that for the swimmer, the smooth body and caudal fin together formed its BCF module, and the paired pectoral fins constituted its MPF module.

**Figure 2:**
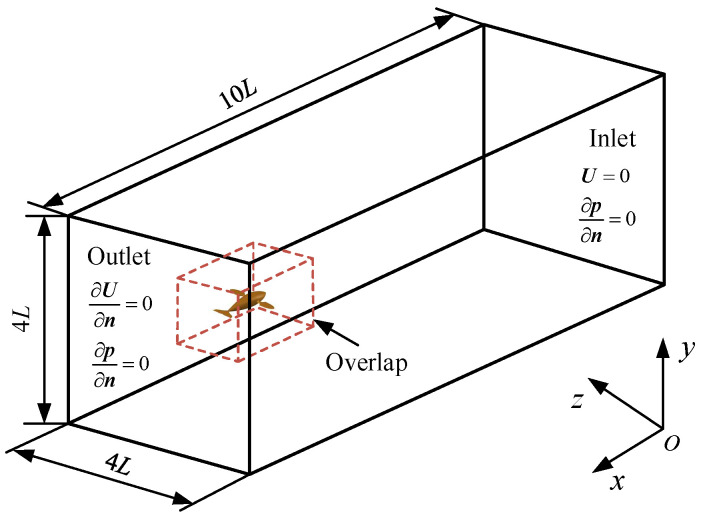
Physical model of the computational domain.

The computational domain around the swimmer was a 3D cubic water tank with a size of 10*L* × 4*L* × 4*L* containing a small body-fitted space with a size of 2.4*L* × 1.2*L* × 1.2*L* (a dotted cubic box), as shown in Figure 2. The body-fitted space and the other fluid domain were connected by the overlapping grids. The swimmer was placed 1*L* from the outlet, at the center of the plane formed by the lateral and vertical directions. Enough space was left longitudinally so that the swimmer could achieve self-propulsion. It should be noted that a right-handed absolute coordinate system *oxyz* was also established, as shown in Figure 2, where the *ox* axis pointed to the tail, the *oy* axis pointed to the top and the *oz* axis pointed to the side. The velocity inlet and pressure outlet constituted the boundary conditions of the incoming flow. The velocity imposed on the upstream boundary was zero, and the velocity gradient imposed on the downstream boundary and other boundaries was zero. In addition, the pressure gradient of all boundaries was zero.

**Figure 3:**
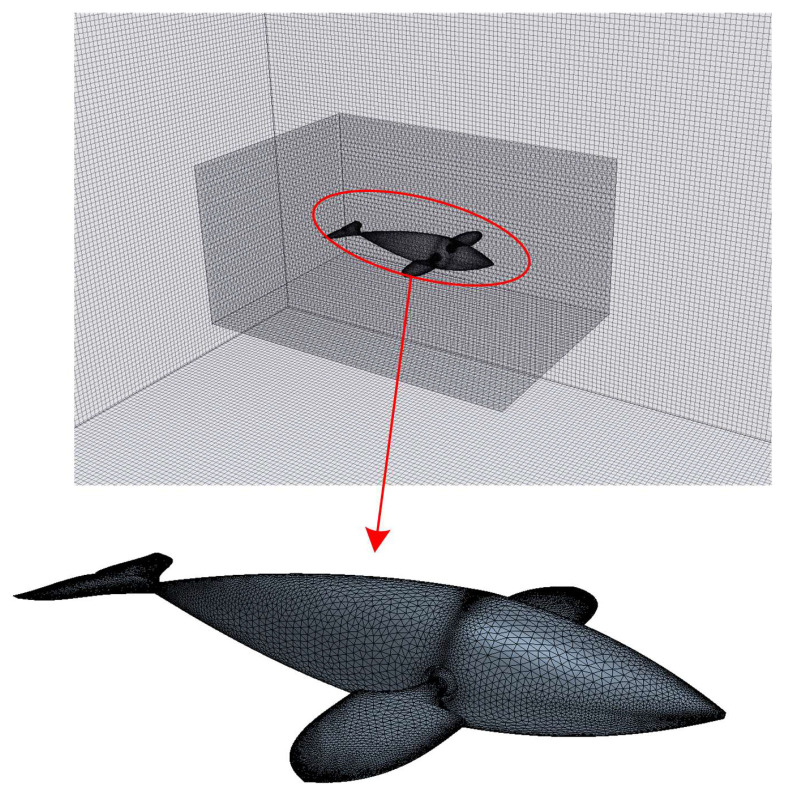
Specific details of the overlapping grids division.

The entire computational domain contained nearly 5.2 × 10^6^ structured grids and 0.6 × 10^6^ unstructured grids. Among them, the background domain used the structured grid to deal with the flow problems, and the body-fitted space used the unstructured grid to discretize in order to deal with the large-scale swing of multiple fins and the flexible deformation of the body, which were validated in previous studies [15,39,40]. During the whole self-propulsion process, the body-fitting space moved with the swimmer. The surface of the swimmer was discretized into a triangular grid with a side length of 0.001*L*; the surface of the small cubic box was also discretized into a triangular grid with a side length of 0.002*L*, and the surface of the outer watershed was divided into a rectangular grid with a side length of 0.0025*L*. Figure 3 shows the specific details of the overlapping grid division in this work. It is important to note that the grid must be locally refined near the swimmer area in the body-fitted space to ensure the reliability of the solution process. Different grid division schemes and solving algorithms were used in different subregion spaces, which not only guarantees computational accuracy but also greatly improves computational efficiency.

### 2.2. Kinematics

**Figure 4:**
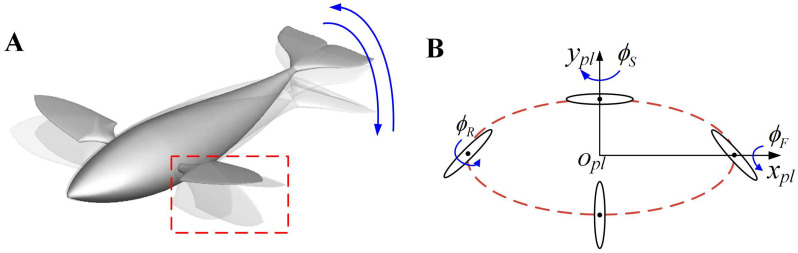
Schematic diagram of swimmer’s multi-fin movement. (**A**) Decomposition diagram of composite motion of swimmers during one cycle; (**B**) The discrete pattern of the pectoral fin motion.

The kinematics of the swimmer can be divided into two aspects: one is the up-and-down wave of the body and compound swinging of the caudal fin, and the other is the paddling motion of pectoral fins. The schematic diagram is shown in Figure 4. Strictly, the regular movements of the body and caudal fin constitute the BCF mode, while the symmetrical pectoral fin movements form the MPF mode. The up-and-down wave of the body can be abstracted as a traveling wave propagating from the head to the tail, described as

(1) $$y(x,t)=(C_{0}+C_{1}x+C_{2}x^{2}+C_{3}x^{8})\cdot A_{\max}\cdot \sin[2\pi (\mathrm{ft}-\frac{x}{\lambda })]$$

where *y*(*x*, *t*) represents the flexible displacement of the body, *A*_max_ is the amplitude of the trailing edge of the body along the *y* axis and has a value of 0.1*L*, *x* is the *x* axis coordinate, *t* is the time, *f* is the body wave frequency and *λ* is the body wavelength. Specially, the amplitude envelope function is composed of amplitude coefficients *C*_0_, *C*_1_, *C*_2_ and *C*_3_, where *C*_0_ = 0.21, *C*_1_ = −0.66, *C*_2_ = 1.1 and *C*_3_ = 0.35. The kinematics of the caudal fin consist of a heaving motion and a pitching motion, which can be expressed as

(2) $$\left\{ \begin{array}{l} y_{\mathrm{cf}}(t)=A_{\max}\sin[2\pi (\mathrm{ft}-\frac{L_{b}}{\lambda })]\\ \theta (t)=\theta_{\max}\sin[2\pi (\mathrm{ft}-\frac{L_{b}}{\lambda })-\varphi ] \end{array}\right.$$

where *y_cf_*(*t*) denotes the sinusoidal movement of the heaving motion, *θ*(*t*) denotes the angular rotation of the pitching motion, and *θ*_max_ is its maximum amplitude where *θ*_max_ = 45°. *L_b_* is the body length measured from the head to the caudal peduncle of the swimmer, and *φ* denotes the phase difference of the heaving motion ahead of the pitching motion and has a value of 90°.

The paddling motion of the pectoral fins constitutes the other propulsion mode. The basic kinematics of the paddling motion can be described as a compound movement in three aspects: forward-and-backward stroke *ϕ_S_*, up-and-down flap *ϕ_F_*, and roll *ϕ_R_*, which can be depicted as

(3) $$\{\begin{array}{c} \phi_{R}=\phi_{R0}-\phi_{R\max}\cos(2\pi \mathrm{ft})\\ \phi_{S}=\phi_{S0}-\phi_{S\max}\cos(2\pi \mathrm{ft}+\Delta \phi_{S})\\ \phi_{F}=\phi_{F0}+\phi_{F\max}\cos(2\pi \mathrm{ft}+\Delta \phi_{F}) \end{array}$$

where *ϕ_R_*_max_, *ϕ_S_*_max_ and *ϕ_F_*_max_ denote the maximum amplitudes of the three angles, respectively, and *ϕ_R_*_0_, *ϕ_S_*_0_ and *ϕ_F_*_0_ are their initial values (where *ϕ_R_*_max_ = 0, *ϕ_S_*_max_ = 20°, *ϕ_F_*_max_ = 10°, *ϕ_R_*_0_ = π*t*/*T*, *ϕ_S_*_0_ = 0 and *ϕ_F_*_0_ = 0). Δ*ϕ_S_* and Δ*ϕ_F_* represent the phase difference between the stroking angle or the flapping angle and the rolling angle, respectively, where Δ*ϕ_S_* = −90° and Δ*ϕ_F_* = −180°. Figure 4 shows a more vivid paddling behavior of the pectoral fins during one cycle.

### 2.3. Kinematics

To realize the self-propelled collaborative movement of the swimmer, the Navier–Stokes momentum conservation equation and mass conservation equation were adopted as the fluid governing equations in the numerical simulation, which can be expressed as

(4) ∇⋅u=0

(5) $$\rho \frac{\partial u}{\partial t}+\rho (u\cdot \nabla )u=-\nabla p+\mu \nabla^{2}u$$

where ∇ is the gradient operator, ***u*** is the fluid velocity vector, *ρ* is the fluid density, *p* is the pressure divided by the density and *μ* is the dynamic viscosity. To simulate the numerical variations of the fluid flow surrounding the virtual swimmer, a nonslip boundary condition needed to be enforced on the mobile interface, with the velocity of the fluid nodes $\dot{\zeta }$ and the velocity of the surface nodes of the swimmer $\dot{x}$ written as

(6) $$\dot{\zeta }=\dot{x}$$

Newton’s equations of motion were adopted as the governing equations for the spatial motion of the swimmer in the instantaneous iteration process, described as

(7) $$m\ddot{X}=F$$

where ***F*** is the fluid force vector acting on the swimmer, *m* is the mass of the swimmer and $\ddot{X}$ is the forward acceleration vector of the swimmer. From the perspective of hydrodynamics, the fluid force vector can be computed as

(8) $$F=\oint_{S}\sigma \cdot n\;dS$$

where ***σ*** is the stress tensor, ***n*** is the unit vector along the normal direction and *dS* is the differential unit area of the swimmer’s surface.

### 2.4. Numerical Method and Validation Test

In terms of the numerical simulation, the commercial software FLUENT with pressure-based transient solvers was chosen to solve the fluid dynamics problems. The unsteady flow field was effectively simulated by the efficient method of decomposing the computational domain and solving the governing equations in parallel. The active deformation of the swimmer was realized through the internal DEFINE_GRID_MOTION macro, and the DEFINE_CG_MOTION macro was used to realize the self-propelled forward movement of the virtual carrier. The proposed method was based on Newton’s equations of motion and applied the user-defined functions and the overlapping grid technique to both modes of active deformation and overall passive propulsion of the swimmer. In the solution process, the finite volume method was used to discretize the Navier–Stokes equation, in which the gradient interpolation was based on the Green–Gauss element, the convection term adopted a second-order upwind scheme, and the diffusion term adopted a second-order central differential scheme. The pressure–velocity coupling of the continuity equation was implemented using the SIMPLE algorithm.

All simulations were carried out with constant viscosity of water, i.e., *μ* = 1.01 × 10^−3^ Pa∙s, and the fluid density was *ρ* = 1.0 × 10^3^ kg∙m^−3^. Since the swimmer was undergoing self-propulsion, the swimming velocity was not prescribed first and increased gradually from zero to a steady value. The resulting Reynolds number (*Re*) based on the swimming velocity also gradually increased from zero to a steady-state value, which is limited to a certain range to meet the needs of the simulation environment. In addition, it should be noted that the size of the swimmer here was consistent with that of the robotic prototype developed in the laboratory, and a shorter length was used. Based on the calculations that showed the swimming velocity increasing from zero to the steady value and the unchanging length of the swimmer, *Re* was roughly between (*Re*~0) and (*Re*~10^4^). Within this scope of continuous variation of *Re*, it is very difficult to distinguish clearly how much the critical *Re* number was. The flow regimes for all self-propulsion processes are difficult to undergo; for some, this may be for laminar flow, and for some, turbulent flow. In this condition, a feasible approach might be the use of the transitional regime to conduct system research.

**Figure 5:**
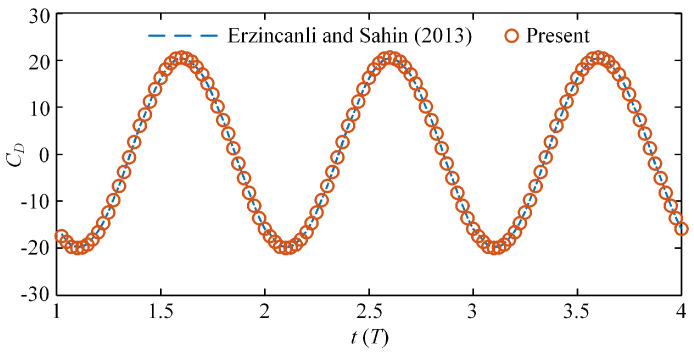
Time history of the related drag coefficient from the present calculations (red ball line) compared with the results of Erzincanli and Sahin (blue dashed line).

**Figure 6:**
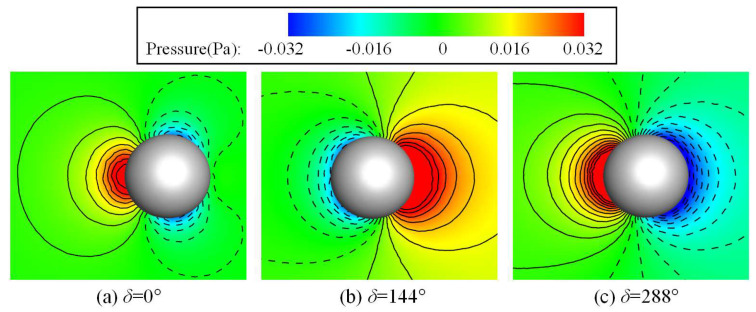
Contours of pressure at three different phase angles.

To verify the validity of the numerical method to study the comparison and collaboration of the two swimming modes in dolphins, we chose the oscillation case of a 3D sphere and calculated two sets of cases with different parameters, one identical to the work of Erzincanli and Sahin [41], and the other identical to the literature Ref. [42], in order to double-verify the accuracy of the numerical method. In the first case, the motion of the sphere was prescribed as $x_{c}(t)=A_{m}[1-\cos(2\pi f_{s}t)]$, where *A*_m_ = 0.125*D* is the oscillating amplitude, *D* is the diameter of the sphere, *f_s_* = 1 Hz is the oscillating frequency and the Reynolds number is taken to be *Re* = 20, which is the same as was used in the work of Erzincanli and Sahin. For the drag coefficient, the calculation method was the same as that in the literature, namely, $C_{D}=4F_{x}/(1/2)\rho \pi D^{2}U_{\max}^{2}$, where *F_x_* is the fluid force along the horizontal *x* direction and *U*_max_ is the maximum speed of the sphere. Figure 5 shows the time history variations of the drag coefficients calculated in this paper and a comparison with the results of Erzincanli and Sahin [42]. The results show that the drag coefficients calculated by our method are almost consistent with the results in Erzincanli and Sahin’s work. In the second case, the motion of the sphere was defined as $x_{c}(t)=A_{m}\sin(2\pi f_{s}t)$, where *A*_m_ = 0.125*D* and *f_s_* = 1.2732*U*_max_/*D*. It is necessary to note that in this case, the Reynolds number was 78.54, based on the sphere diameter *D* and the maximum speed of the sphere *U*_max_, and the Strouhal number was 1.2732. Figure 6 shows the pressure contours at three different phase angles, which are also in high agreement with the results reported in the literature to double-verify the accuracy of the numerical method in this work [42].

**Figure 7:**
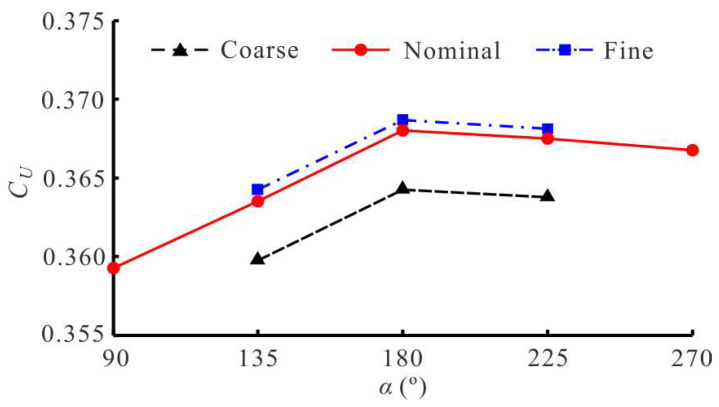
Convergence test of three different sizes of grid.

A grid convergence test was also performed to determine the appropriate discretization size. In this work, three grid sizes with uniform side lengths of 0.004*L*, 0.002*L* and 0.001*L* were used, and the corresponding grid numbers were 1.2 million (coarse), 5.8 million (nominal) and 12.5 million (fine), respectively. For each grid, the domain size, time step and boundary conditions were the same as those used for the nominal grid, which is the grid used for all simulation cases. In the quantitative grid convergence test, we unified the other kinematic parameters of the swimmer except for the starting phase difference α between the BCF and MPF modes, and took the value of *α* as 135°, 180° and 225°. Figure 7 shows the dimensionless steady-state swimming velocity *C_U_* of three different grids as a function of *α*. It can be observed that under the same conditions, the swimming law simulated by the coarse grid had a large error, while the nominal grid and the fine grid could better deal with the hydrodynamics of multimode collaborative cases. Therefore, the nominal grid with a uniform size of 0.002*L* was a suitable choice for this study.

### 2.5. Calculation of Performance Parameters

In this work, we focused on the comparison and collaboration of the BCF and MPF modes and applied several parameters to quantitatively evaluate the propulsive performance. Firstly, the instantaneous fluid force components along the *x* and *y* axes was calculated from the pressure and viscosity components acting on the swimmer, written as

(9) $$F_{x}(t)=\int_{S}-pe_{1}\mathrm{dS}+\int_{S}\tau_{1j}e_{j}\mathrm{dS}$$

(10) $$F_{y}(t)=\int_{S}-pe_{2}\mathrm{dS}+\int_{S}\tau_{2j}e_{j}\mathrm{dS}$$

where *e_j_* is the *j*th component of the unit normal vector on *dS* (i.e., *j* = 1, 2, 3 correspond to *x*, *y*, *z* directions in the absolute coordinate system, respectively) and *τ_ij_* is the viscous stress tensor. To facilitate describing the contribution of each mode, the swimmer’s propulsion mode was divided into the BCF mode and the MPF mode, and their instantaneous fluid force components along the *x* axis were denoted as *F_xbcf_*(*t*) and *F_xmpf_*(*t*), respectively, so that the overall fluid force was written as *F_x_*(*t*) = *F_xbcf_*(*t*) + *F_xmpf_*(*t*). The calculation method for these two components *F_xbcf_*(*t*) and *F_xmpf_*(*t*) was consistent with Equation (9). For the method of solving the fluid force components generated by different modes, we needed to choose the corresponding force surface. On this basis, the average fluid force produced by the BCF mode was defined as ${\bar{F}}_{XBCF}$, and the average fluid force produced by the MPF mode was defined as ${\bar{F}}_{XMPF}$. In this way, the average fluid force of the swimmer along the x axis ${\bar{F}}_{X}$ was expressed as ${\bar{F}}_{X}={\bar{F}}_{XBCF}+{\bar{F}}_{XMPF}$.

Note that during the swimmer’s self-propulsion, both the pressure and viscosity components of the fluid force changed periodically, and their contributions to thrust and resistance were judged from the change in sign of the fluid force *F_x_*(*t*). Therefore, the thrust *F_T_*(*t*) and resistance *F_R_*(*t*) were decomposed into the following equations.

(11) $$F_{T}(t)=\frac{1}{2}\left(\int_{S}-pe_{1}\mathrm{dS}+\left|\int_{S}pe_{1}\mathrm{dS}\right|\right)+\frac{1}{2}\left(\int_{S}\tau_{1}{}_{j}e_{j}\mathrm{dS}+\left|\int_{S}\tau_{1}{}_{j}e_{j}\mathrm{dS}\right|\right)$$

(12) $$F_{R}(t)=\frac{1}{2}\left(\int_{S}-pe_{1}\mathrm{dS}-\left|\int_{S}pe_{1}\mathrm{dS}\right|\right)+\frac{1}{2}\left(\int_{S}\tau_{1}{}_{j}e_{j}\mathrm{dS}-\left|\int_{S}\tau_{1}{}_{j}e_{j}\mathrm{dS}\right|\right)$$

In this sense, we redefined the equation of instantaneous fluid force of the swimmer in the *x* axis as *F_x_*(*t*) = *F_T_*(*t*) + *F_R_*(*t*).

Secondly, the power consumed by the swimmer to overcome the fluid reaction force of the up-and-down swing was expressed as $P_{V}(t)=F_{y}(t)\cdot \dot{Y}(t)$. To fully illustrate the self-propulsion performance of the swimmer, the propulsion efficiency based on the Froude method needs to be introduced here, calculated as

(13) $$\eta_{D}=\frac{{\bar{F}}_{T}U}{{\bar{F}}_{T}U+{\bar{P}}_{V}}$$

where ${\bar{F}}_{T}$ is the average thrust force over a full cycle and *U* is the steady swimming velocity along the propulsive direction, i.e., the negative *x* direction. Due to the up-and-down swing movement, ${\bar{P}}_{V}$ is defined as the average power consumption through one cycle.

Finally, to give the numerical solutions obtained in this work universal meaning, several important performance parameters need to be dimensionless and defined as

(14) $$C_{\mathrm{FBCF}}=\frac{{\bar{F}}_{\mathrm{XBCF}}}{0.5\rho U^{2}L^{2}},\;\;C_{\mathrm{FMPF}}=\frac{{\bar{F}}_{\mathrm{XMPF}}}{0.5\rho U^{2}L^{2}}$$

(15) $$C_{\mathrm{FT}}=\frac{{\bar{F}}_{T}}{0.5\rho U^{2}L^{2}}$$

(16) $$C_{\mathrm{PV}}=\frac{{\bar{P}}_{V}}{0.5\rho U^{3}L^{2}}$$

where *C_FBCF_* and *C_FMPF_* are the dimensionless average fluid force component generated by the BCF mode and the MPF mode, respectively, *C_FT_* is the dimensionless average thrust force and *C_PV_* is the dimensionless power loss. For the dimensionless representation of the swimming velocity, we applied *C_x_* and *C_U_* to denote the dimensionless instantaneous swimming velocity and the dimensionless average swimming velocity in a cycle, respectively.

(17) $$C_{x}=\frac{\dot{X}(t)\cdot T}{L},\;\;C_{U}=\frac{U\cdot T}{L}$$

where $\dot{X}(t)$ is the instantaneous swimming velocity along the negative *x* direction in the absolute coordinate system. It should be noted that Strouhal number (*St*) is a very important dimensionless parameter commonly used to measure the efficiency of the swimmer and can be defined as

(18) $$\mathrm{St}=\frac{f\cdot 2A_{\max}}{U}$$

## 3. Results and Discussion

In this section, we will focus on comparing the propulsion performance of the swimmer in single mode and in collaborative mode. The kinematic parameters of the BCF and MPF modes referred to Equations (2) and (3), respectively. Here, we set the swing frequency to 5 Hz and used it as a basis to study the variation of the frequency ratio. The feasibility of the swimmer under self-propulsion and the potential superiority of the collaboration mode were obtained through the results and discussion of different situations.

### 3.1. Time History Variations of Performance Parameters

**Figure 8:**
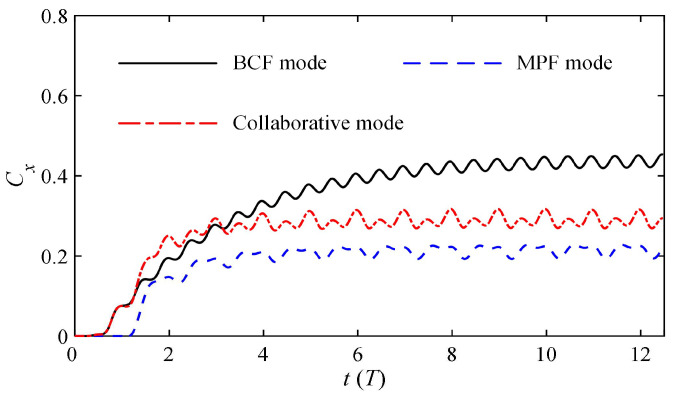
Time history curves of the transient swimming velocity with different modes.

The virtual swimmer experienced a dynamic and gradual convergence process from stationary to a steady-state cruise. The collaborative mode of the swimmer was defined as the combination of the BCF and MPF modes mentioned above. This section mainly compares the propulsion effects produced by several different modes. Figure 8 shows the time history of the dimensionless instantaneous swimming velocity *C_x_* obtained at the same frequency in BCF mode, MPF mode and collaborative mode, respectively. The results show that the MPF mode based on the paired pectoral fins produces the lowest steady velocity with a faster convergence acceleration, while the BCF mode with the body and caudal fin module produces the largest steady velocity at the same frequency. The final cruise speed was converted to 2.25*BL*/*s* in measurement unit of body length (*BL*), which meets the swimming speed range of normal dolphins in nature [43]. The most difficult to analyze are the variation trends produced by the collaborative mode. In the initial stage of the startup, the collaborative explosive force of the two modes has the greatest acceleration and quickly converges. However, at the inflection point after convergence, the steady velocity gradually stabilizes, which is slower than that of single BCF-mode propulsion. From this point, it can be inferred that on the basis of the BCF mode, the MPF mode produces resistance rather than thrust when the swimmer enters the steady state. Therefore, we wondered if the resistance effect of the MPF mode could be converted into a thrust effect, and if so how it could be converted. These are two very enlightening questions.

**Figure 9:**
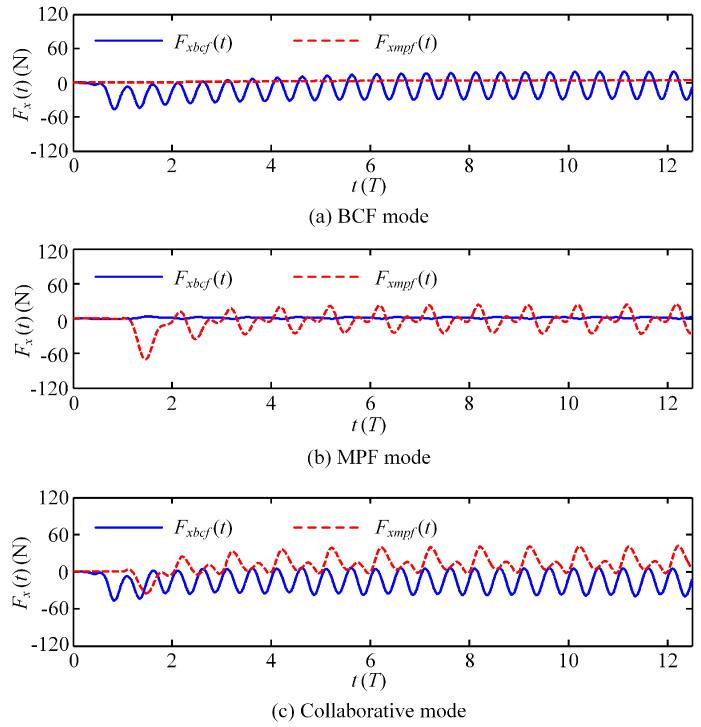
Fluid forces generated by the two modules under three different movement modes.

To explain the reason why the steady velocity of the collaborative mode is lower than that of the single BCF mode, Figure 9 shows the convergence of the fluid forces generated by three different modes along the advancing direction, i.e., the negative *x* axis direction. Obviously, in a single mode, that is, the BCF mode or the MPF mode, the resistance of the static part of the swimmer is greater than zero and fluctuates in a small range. The working part plays a role in accelerating the propulsion, and thus, the generated fluid force gradually fluctuates around a negative value near zero after reaching the steady state, corresponding to the velocity curve of the swimmer moving forward smoothly, as shown in Figure 8. For the collaborative mode, both the BCF and MPF modules play a positive role in the initial stage. However, after reaching the stable stage, the fluid force generated by the MPF module gradually becomes resistance, which exactly explains the reason why the steady velocity of the collaborative mode is lower than that of the single BCF mode under the same frequency. From this, it can be inferred that stacking the MPF module is not the best way to improve the propulsion performance of the swimmer in collaborative mode. On the contrary, swimmers in MPF mode have better maneuverability and stability than those in BCF mode. Therefore, we believe that the MPF module has more advantages in the 3D maneuvering motion of swimmers, which is the direction of our future research.

### 3.2. Effect of Starting Phase Difference on Self-Propelled Performance

**Figure 10:**
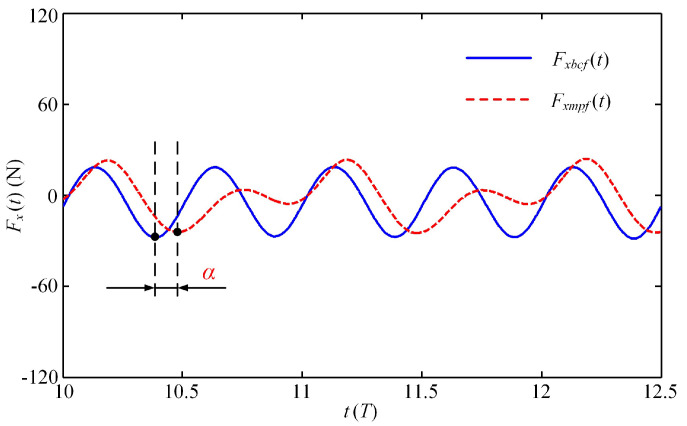
Transient fluid forces generated by the two modules in the steady state.

Obviously, the BCF and MPF modes play different roles in the swimmer’s propulsion. To distinguish their respective contributions, Figure 10 shows the instantaneous fluid force generated by these two modes after the swimmer reaches the steady state. It can be observed that in the MPF mode, the swimmer reaches the maximum thrust at *n* + 0.5*T*, while in the BCF mode, the maximum thrust value of the swimmer occurs at both *n* + 0.4*T* and *n* + 0.9*T*, which means that in the BCF mode, the swimmer can alternately generate the maximum thrust twice in one cycle, where *n* denotes the *n*-th movement cycle in the self-propulsion process. It is necessary to note that when the instantaneous fluid force reaches a negative maximum value, it is considered to have reached the maximum thrust force at this time. For the convenience of description, here we define the time interval between the two modes to generate the maximum thrust value in a single cycle as the starting phase difference *α*, in which the BCF mode is activated prior to the MPF mode. The influence of the starting phase difference *α* on the propulsion performance in the collaborative mode is further discussed below.

**Figure 11:**
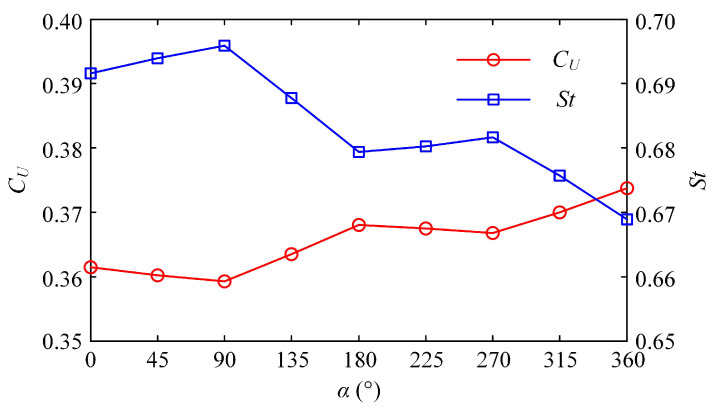
Effect of the starting phase difference *α* on the dimensionless steady-state swimming velocity *C_U_* and the Strouhal number *St*.

To answer the two questions posed above, we tried to change the starting phase difference between the BCF and MPF modes to improve the collaborative effect of the latter. Figure 11 shows the effect of the starting phase difference *α* on the dimensionless steady-state swimming velocity *C_U_* and the Strouhal number *St*. The entire curve of *C_U_* presents an upward retracement trend and eventually stabilizes, while the law of *St* curve is the opposite. Careful observation shows that when the starting phase difference is a multiple of 180°, the dimensionless steady-state velocity *C_U_* reaches its maximum value, while *St* is at the trough value during one cycle. At this point, both the BCF and MPF modes are at the moment when they generate the maximum thrust. In both half-cycles, the value of *C_U_* first linearly decreases with the increase of *α*, then linearly increases after reaching the inflection point. With the continuous increase of *α*, it means that the starting time of the MPF mode is continuously delayed, with the result that the pectoral fins in the acceleration phase will not provide too much resistance; therefore, the value of *C_U_* will gradually increase and converge to the maximum steady-state velocity generated by the BCF mode. Regarding the variation of the *St* number, the overall fluctuation range of its value is between 0.6 and 0.7. With the continuous increase of *α*, the value of *St* gradually decreases and approaches stability, while *C_U_* changes in the opposite direction. The law of the two complementary changes is consistent with the experimental conclusions on the oscillating wing [44].

**Figure 12:**
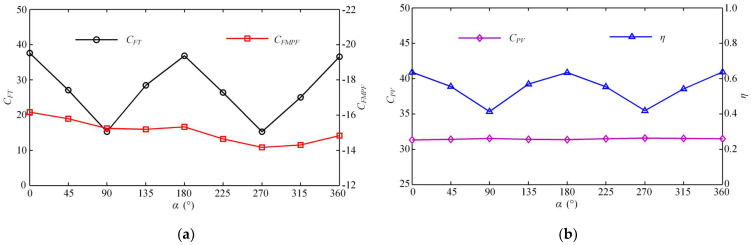
Relation between the thrust force, power loss, efficiency and starting phase difference α. (**a**) Relation between *C_FT_*, *C_FMPF_* and *α*. (**b**) Relation between *C_PV_*, $\eta_{D}$ and *α*.

Furthermore, Figure 12a shows the variation of the average thrust force coefficient *C_FT_* with the starting phase difference *α* after the swimmer enters the steady state. As *α* increases gradually, *C_FT_* fluctuates up and down. When *α* is a multiple of 180°, the BCF and MPF modes that generate the maximum thrust can create better resonance. The value of *C_FT_* at this time reaches its largest. At the moment when *α* is a multiple of 90°, the work of the two modes does not collaborate well, and the propulsion effect produced by their combination is relatively weak. It should be noted that in our simulation, the swimmer’s body and caudal fin constitute the BCF module, and a pair of pectoral fins constitutes the MPF module, each with its own motion mode. Figure 12a also shows the average fluid force coefficient produced by the MPF mode in the steady state. It can be seen that at the same frequency, the BCF mode plays a significant role in propulsion, while the MPF mode does not meet our propulsion expectations but plays a role of resistance. It is consistent with the time history of the fluid forces generated by the MPF mode shown in Figure 9c. The change rule of the starting phase difference shows that as the starting time of the pectoral fins is delayed, the resistance generated by the MPF mode decreases. This result indirectly confirms that the lagging effect of the MPF mode at the same frequency helps to increase the steady-state swimming velocity of the later cruise.

The change laws of energy loss coefficient *C_PV_* and propulsive efficiency $\eta_{D}$ calculated according to the Froude theory are shown in Figure 12b. Under the same frequency, *C_PV_* is not significantly affected by the change of starting phase difference *α*, while the fluctuation of propulsive efficiency is relatively large, and the law of curve change is similar to that of *C_FT_* in Figure 12a. When *α* is a multiple of 180°, the BCF and MPF modes that produce the maximum thrust at the same time can form a better resonance, and thus the propulsive efficiency at this time also reaches the largest. When *α* is a multiple of 90°, the propulsive efficiency produced by the two modes is the lowest.

### 3.3. Effect of Frequency Ratio on Self-Propelled Performance

**Figure 13:**
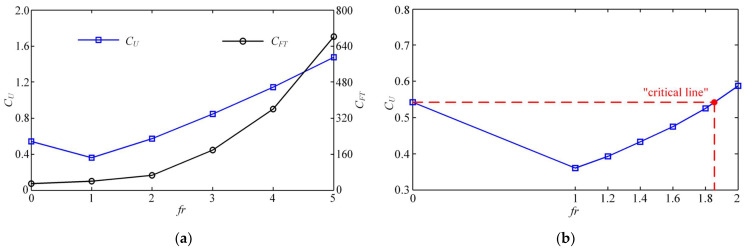
(**a**) Relation between the dimensionless steady-state propulsion velocity *C_U_*, overall force coefficient *C_FT_* and frequency ratio *β*; and (**b**)definition of “critical line”.

In addition, to explore the potential superiority of the collaboration between the two modes and reduce the adverse impact of MPF mode on propulsion, we tried to increase the stroke frequency of MPF mode and kept the swing frequency of BCF mode constant. Here, the frequency ratio *β* of MPF mode and BCF mode is defined to describe the collaborative work of high-frequency MPF motion. Figure 13a shows the variations of dimensionless steady-state velocity *C_U_* and overall thrust force coefficient *C_FT_* with the frequency ratio *β* changing from 0 to 5. With the increase of *β*, *C_U_* increases slowly, and *C_FT_* increases with a steeper growth trend after gradually increasing. It is necessary to explain that when *β* = 0, the swimmer is in the state of single BCF propulsion. Closer observation of Figure 13a shows that *C_U_* when *β* = 2 slightly exceeds the value of *C_U_* when *β* = 0, and the stroke of the MPF mode becomes beneficial at this time. As *β* further increases, the value of *C_U_* rises linearly, which proves that the dual-mode collaboration with a certain frequency ratio can obtain a better propulsion effect. This is a very interesting phenomenon that has not been reported elsewhere.

To further reveal the superposition effect produced by the collaborative mode at different frequency ratios, we subdivided the values of *β* ranging from 1 to 2 to obtain a “critical line”, after which the dual-mode collaboration became favorable. As shown in Figure 13b, when *β* is between 1.8 and 2, the dimensionless steady-state velocity *C_U_* of the swimmer with collaborative mode can reach the value of the swimmer with single BCF propulsion. In other words, when *β* is higher than the “critical line” shown in Figure 13b, the existence of the MPF mode actually facilitates the propulsion, and when it is below the “critical line”, the MPF mode keeps hindering the swimmer’s advancement.

**Figure 14:**
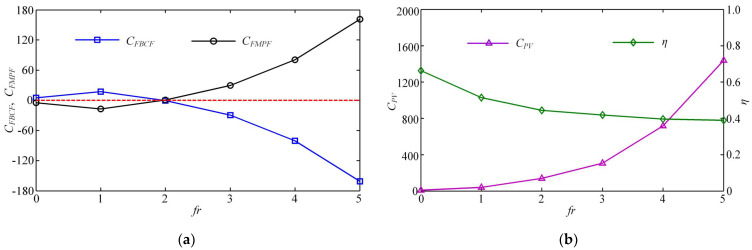
(**a**) Relation between the dimensionless thrust force and frequency ratio *β*. The red dashed line represents the baseline with a value of 0. (**b**) Relation between the power loss, efficiency and frequency ratio *β*.

Moreover, Figure 14a shows the dimensionless fluid force components produced by each of the two modes for different frequency ratios. Since the swimmer moves at a constant velocity, i.e., the net fluid force is zero, the fluid force components produced by the two modes are mutually constrained. It can be observed from Figure 14a that in the case of *β* = 1, the MPF mode plays a role in resistance, thus the swimming velocity is not as obvious as the effect caused by the single BCF propulsion. In this sense, the MPF mode for stroking is a burden during the swimmer exercise at this time. When *β* = 2, the dimensionless fluid force components generated by these two modes are almost zero in the steady state, and thus the MPF and BCF modes have reached a certain balance at this time. Finally, as the frequency ratio continues to increase, the effect of the MPF mode will become more obvious, and the value of *C_FMPF_* increases with a steeper growth trend. However, the BCF mode that once played a propulsive role at low *β* has turned from a positive propulsion to a burden that generates resistance.

To reveal the mapping relationship between the dual-mode collaboration frequency ratio and the energy parameters, we plotted the average power loss coefficient *C_PV_* and the propulsive efficiency $\eta_{D}$ as a function of *β*, as shown in Figure 14b. With the increase of *β*, *C_PV_* increases with a steeper growth trend after gradually rising, and $\eta_{D}$ gradually slows down and becomes stable after a sharp decline. When the swimmer reaches the high-frequency ratio, although the steady swimming velocity increases, the propulsive efficiency is not as good as the value in the low-frequency ratio case.

### 3.4. Three-Dimensional Flow Structure

**Figure 15:**
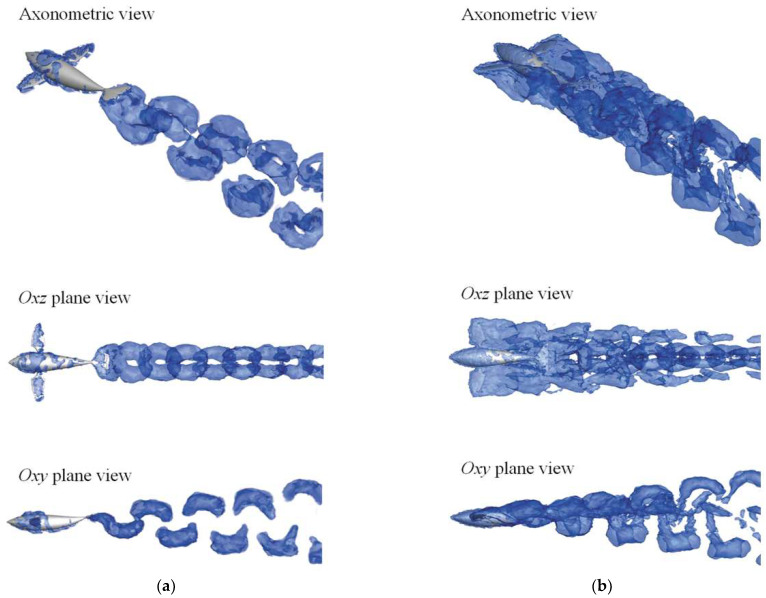
The vorticity isosurfaces of the two motion modes: (**a**) BCF mode (*β* = 0); and (**b**) collaborative mode (*β* = 1).

In this section, we further characterize the 3D flow structure formed by different modes of the swimmer. To facilitate the distinction, Figure 15 extracts the vorticity isosurfaces of the BCF mode (*β* = 0) and collaborative mode (*β* = 1) based on the *q* criterion, which is presented in three perspectives, respectively. The value of *q* is defined as *q* = (║*Ω*║^2^ − ║*S*║^2^)/2, where *S* and *Ω* denote the symmetric and asymmetric tensors of the velocity gradient, respectively, and ║·║ is the Euclidean matrix norm. In the region where the velocity dominates the strain rate (i.e., *q* > 0), it is occupied by the vortex structure [45]. Visualization of the fluid shows the vortex structures that are continuously shed from the trailing edge along the propulsive direction, where the vortices around the swimmer are stacked.

Closer inspection of Figure 15 shows that the distribution of vortices generated by the BCF mode is consistent with that of the collaborative mode, except for the vortices generated by the pectoral fins. When *β* = 0, i.e., BCF mode, the pectoral fins on both sides of the swimmer are at rest, and a small number of vortices are attached to the surface during self-propulsion. The shape of the vortex streets in the wake has a regular pattern, and the “hairpin” vortex streets are distributed in double rows in the downstream, and gradually dissipate to the rear. When *β* = 1, i.e., collaborative mode, the vortices generated by the two modules cannot be well separated, but it can be found that the overall vortex structure is composed of four rows of vortex streets extending backward. The “hairpin” vortex streets that spread up and down in the *oxy* plane are generated by the periodic swing of the BCF module, while the symmetrical stroke of the MPF module in the *oxz* plane also generates two rows of vortex streets toward the wake region. Unlike the vortex streets generated by the BCF module that spread up and down, the two columns of vortex streets generated by the MPF module spread to the left and right, respectively, and their diffusion rate is also significantly faster than the other two columns.

**Figure 16:**
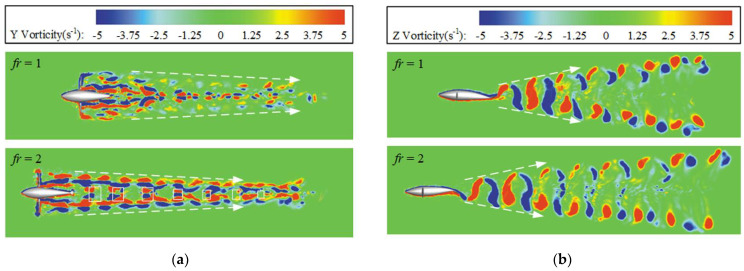
The vorticity contours of multifin mode within two sections: (**a**) *oxz* plane view; and (**b**) *oxy* plane view.

Since the 3D isosurface cannot well express the vortex structure in the collaborative mode, here we select the *oxy* and *oxz* cross-sections as the projection planes to study the vortex laws generated by the BCF and MPF modules, respectively. As shown in Figure 16a, in the *oxz* cross-section, when *β* = 1, the wake area is relatively messy, and the vortices generated by the two modules are clustered toward the rear, showing an irregular distribution. This is due to the fact that the MPF module acts as a resistance, causing the periodic strokes of the pectoral fins to continuously disperse the vortices attached to the swimmer. When *β* = 2, it can be seen that in the two rows of deep vortex streets formed by the paired pectoral fins, the width of the vortex streets gradually becomes narrower, and the vortex streets formed by the caudal fin are constantly squeezed. The white rectangular box shows a pair of vortex structures periodically formed by the BCF module. Closer observation of Figure 16a shows that as the distance from the main body of the swimmer increases, the vorticity gradually decreases and eventually dissipates.

As shown in Figure 16b, the *oxy* section can only express the vorticity generated by the BCF module, including two diffuse vortex streets. This conclusion is consistent with the previous wake vortex structure reported in the single BCF propulsion [45,46]. Since the swing of the caudal fin is fixed and the basic frequency is unchanging, the value of frequency ratio *β* does not affect the overall layout of the vortex street generated by the caudal fin. However, as the value of *β* increases, the steady swimming velocity of the swimmer increases, and therefore the longitudinal spacing of the vortex street gradually becomes larger.

## 4. Conclusions

In this work, we systematically synthesized two universal motion modes suitable for dolphins, namely, BCF mode and MPF mode, and conducted comparative and collaborative studies on them. As a result, we have obtained the advantages of dual-mode collaboration and its potential solvable shortcomings. The main findings can be summarized as follows:

(1) Given the same frequency, the swimming process gradually converges, and the propulsion effect of the swimmer in the BCF mode is better than that in the MPF mode and the collaborative mode, which is mainly reflected in the value of the final steady-state swimming velocity and the related thrust force. It was found that the participation of the MPF module does not promote the acceleration of the swimming, but plays a cumbersome role. Fortunately, it was found in this work that there are two ways to improve their collaborative performance; one is to adjust the phase difference between the two modes, and the other is to optimize the frequency ratio between the two modes.
(2) The definition of the starting phase difference *α* is helpful to analyze the superposition effect of the two modules in the collaborative mode. When *α* is a multiple of 180°, the final steady-state velocity reaches the maximum. When *α* is a multiple of 90°, the resistance generated by the MPF mode is relatively large, and the collaborative effect of the two modes is not ideal at this time. The starting phase difference *α* is perhaps the most direct variable to adjust the collaboration of the two modes, and analyzing its quantitative impact is an effective way to explore the contribution of each module to propulsion in the collaborative mode.
(3) It was confirmed that the increase of the frequency ratio *β* can effectively improve the propulsion effect of the MPF mode. When *β* is taken as a critical value between 1.8 and 2, the final steady-state velocity of the swimmer in collaboration mode can reach the value of the swimmer with single BCF propulsion. As *β* further increases, the effect of the MPF mode is more obvious and the value of *C_FMPF_* increases with a steeper growth trend. When the swimmer reaches the high-frequency ratio, the steady-state velocity increases, while $\eta_{D}$ decreases somewhat and is not as good as the low-frequency ratio. These findings suggest that each module of the swimmer contributes unequally to propulsion when multiple modules work together. Therefore, in a sense, it is possible to rationally allocate their contributions to the entire swimmer by adjusting the parameters between the modules, so as to achieve the best collaborative performance, such as the fastest steady-state swimming velocity, the largest thrust or the highest propulsion efficiency.

In summary, a virtual dolphin with BCF and MPF modules was successfully constructed, and the comparison and collaboration of several different modes under self-propulsion were studied. The results of the novel superposition effect prove that only under certain conditions does the MPF mode assisting the BCF mode exert a stronger propulsive function and higher propulsive efficiency. However, if the conditions are not suitable, it can also occur that the two modes hinder each other. In addition, it should be clarified that the current collaborative study is limited to straight-line propulsion. In follow-up research, it is necessary to study the laws of 3D maneuvering motion in space, including steering and pitching, to pave the way for the physical study of biomimetic underwater vehicles.

## Data Availability

All data are available in the main text.
